# *In vitro* co-culture systems for studying molecular basis of cellular interaction between Aire-expressing medullary thymic epithelial cells and fresh thymocytes

**DOI:** 10.1242/bio.201410173

**Published:** 2014-10-17

**Authors:** Yoshitaka Yamaguchi, Jun Kudoh, Tetsuhiko Yoshida, Nobuyoshi Shimizu

**Affiliations:** 1 Advanced Research Center for Genome Super Power, Keio University, 2 Okubo, Tsukuba, Ibaraki 300-2611, Japan; 2 Laboratory of Gene Medicine, Keio University School of Medicine, 35 Shinanomachi, Shinjuku-ku, Tokyo 160-8582, Japan; 3 Institute for Advanced Sciences, Toagosei Company Limited, Tsukuba, Ibaraki 300-2611, Japan

**Keywords:** autoimmunity, AIRE, Aire^+^ cell lines, central self-tolerance, thymic crosstalk

## Abstract

We previously established three mouse cell lines (Aire^+^TEC1, Aire^+^TEC2 and Aire^+^DC) from the medullary thymic epithelial cells (mTECs) and dendritic cells (mDCs). These cells constitutively expressed “autoimmune regulator (*Aire*) gene” and they exhibited various features of self antigen-presenting cells (self-APCs) present in the thymic medullary region.

Here, we confirmed our previous observation that Aire^+^ thymic epithelial cells adhere to fresh thymocytes and kill them by inducing apoptosis, thus potentially reproducing *in vitro* some aspects of the negative selection of T cells *in vivo*. In this system, a single Aire^+^ cell appeared able to kill ∼30 thymocytes within 24 hrs. Moreover, we observed that ectopic expression of peripheral tissue-specific antigens (TSAs), and expression of several surface markers involved in mTEC development, increased as Aire^+^ cell density increases toward confluency. Thus, these Aire^+^ cells appear to behave like differentiating mTECs as if they pass through the developmental stages from intermediate state toward mature state. Surprisingly, an *in vitro* co-culture system consisting of Aire^+^ cells and fractionated sub-populations of fresh thymocytes implied the possible existence of two distinct subtypes of thymocytes (named as CD4^+^ killer and CD4^−^ rescuer) that may determine the fate (dead or alive) of the differentiating Aire^+^mTECs. Thus, our *in vitro* co-culture system appears to mimic a part of “*in vivo* thymic crosstalk”.

## INTRODUCTION

The mammalian thymus consists of two main compartments, cortex and medulla, that are composed of thymic epithelial cell (TEC) populations. Especially, the cortex is composed of cortical TECs (cTECs) and the medulla is composed of medullary TECs (mTECs). Those TECs share distinct functions. In postnatal thymus, the cortical and medullary compartments are formed under precise cellular process “thymic crosstalk”, which is a bi-directional cellular communication between mTECs and developing thymocytes (van Ewijk et al., 1994; Holländer et al., 1995).

In the cortex, some cTECs expressing distinct major histocompatibility complex (MHC) promote positive selection of immature (CD4^−^CD8^−^) thymocytes that are differentiating to CD4^+^CD8^+^ double-positive (DP) thymocytes. Interaction of T-cell receptor (TCR) on DP thymocytes with MHCs on cTECs triggers differentiation of the thymocytes (Bill and Palmer, 1989; Sha et al., 1988; Zepp and Staerz, 1988). The positively selected DP thymocytes in the cortex are then relocated to medulla.

In the medulla, maturation of DP thymocytes takes place along their thymic export (Takahama, 2006; Anderson et al., 2007). One of the crucial functions of medulla is to establish central self-tolerance by eliminating auto-reactive thymocytes. This negative selection is driven by self-antigen-presenting cells (self-APCs), which are specially selected populations of mTECs and medullary dendritic cells (mDCs).

The self-APCs promiscuously (ectopically) express a wide spectrum of peripheral tissue-specific antigens (TSAs) (Derbinski et al., 2001). The ectopically expressed large repertoires of TSAs are then presented to DP-thymocytes (Gallegos and Bevan, 2004; Ardavín, 1997; Amsen and Kruisbeek, 1998). It should be noted that ectopic expression of TSA genes in self-APCs is regulated, at least in part, by a transcription factor AIRE (autoimmune regulator) that we previously found deficient in the patients with autoimmune disease APECED (also known as APS1; OMIM no. 240300) (Nagamine et al., 1997; Aaltonen et al., 1997).

In the thymus of AIRE-deficient APECED/APS1 patients and of Aire-knockout (KO) mice, ectopic expression of several TSA genes was reduced, leading to impaired negative selection of T cells (Anderson et al., 2002; Ramsey et al., 2002). Moreover, reduced TSA gene expression was found in the mTECs of organ-specific autoimmunity (Gotter and Kyewski, 2004; DeVoss et al., 2006).

Autoimmune disease APECED/APS1 is characterized with a diverse array of clinical features, including functional failures of parathyroid gland and adrenal cortex, a high prevalence of auto-reactive T cells and autoantibodies, and immune destruction of multiple organs (Ahonen et al., 1990; Perniola et al., 2000). However, molecular pathogenesis of APECED/APS1 has not been fully understood. Thus, many questions remain to be clarified; how a single mutation of AIRE gene reflects aberrant expression of TSAs for target organs, how a single transcription factor AIRE regulates ectopic expression of TSAs and how special populations of mTECs/mDCs are selected through thymic crosstalk.

Recently, we established three mouse cell lines (Aire^+^TEC1, Aire^+^TEC2 and Aire^+^DC) that constitutively express *Aire* gene and exhibit most of the expected characteristics of mTECs/mDCs (Yamaguchi et al., 2011). These Aire-expressing cells (Aire^+^ cells) adhered to fresh thymocytes and induced apoptosis as if they mimic *in vivo* negative selection of T cells. Moreover, these Aire^+^ cells possessed characteristic features of self-APCs, exhibiting abilities to express a variety of peripheral TSAs, and several essential components of IPSM (immunoproteasome), IS (immunological synapse) and some TNFSFs (tumor necrosis factor super families).

In this study, we made several new findings such as that various critical cellular parameters elevated as Aire^+^cell density increased (semi-confluency *vs* confluency: sparse cells *vs* dense cell–cell contacted cells). We postulated that these Aire^+^ cells in culture may mimic *in vivo* differentiation process of mTECs/mDCs. Moreover, our *in vitro* co-culture system consisting of fractionated thymocytes and Aire^+^ cell lines implied possible existence of two distinct subtypes of thymocytes that may control the fate (dead or alive) of differentiating Aire^+^ cells. We will present the detailed intercellular interaction data to support these notions and the usefulness of Aire^+^ cell lines for *in vitro* study on “thymic crosstalk” will be discussed.

## MATERIALS AND METHODS

All animal experiments were performed in accordance with animal welfare regulations of Laboratory Animal Center, Keio University School of Medicine.

### Cell lines and isolation of mRNAs

Three lines of Aire^+^ cells (Aire^+^TEC1, TE2 and DC) were established as described previously (Yamaguchi et al., 2011). Those Aire^+^ cells (1×10^6^ cells) were seeded in a 90-mm dish (SUMILON) containing DMEM-high glucose medium supplemented with 10% FBS, 100 units/ml penicillin and 100 units/ml streptomycin.

Those cells were grown at 37°C in 5% CO_2_ for 32 hrs to get semi-confluent cultures (0.35×10^7^ cells) and for 72 hrs to get confluent cultures (1×10^7^ cells). Aire^+^TEC1 cells that overexpress FLAG-Aire fusion protein was produced by transfecting plasmid (p3×FLAG/Aire cDNA) as previously described (Yamaguchi et al., 2011). For a negative control of western blotting, mouse A9 skin fibroblast was used. Total RNAs were extracted from Aire^+^ cells using TRIzol reagent (Invitrogen). mRNA was prepared from total RNA using FastTrack MAG Maxi mRNA Isolation Kit (Invitrogen).

### 1^st^ cDNA synthesis and quantitative Reverse Transcription-PCR (qRT-PCR) analysis

Synthesis of 1^st^ cDNA was carried out by reverse transcription from purified mRNA (0.5 µg) using Superscript III kit (Invitrogen) with oligo (dT_20_) and random hexamer primer (Roche). qRT-PCR was performed by TaqMan method with Mouse Universal Probe Library Set (Roche), primers for various genes (Tables 1 and 2) and Fast Star Universal Probe Master (ROX) (Roche) on ABI PRISM 7700 Sequence Detection System (Applied Biosystems). Amounts of specific mRNAs were normalized to β-Actin mRNA.

**Table 1.**
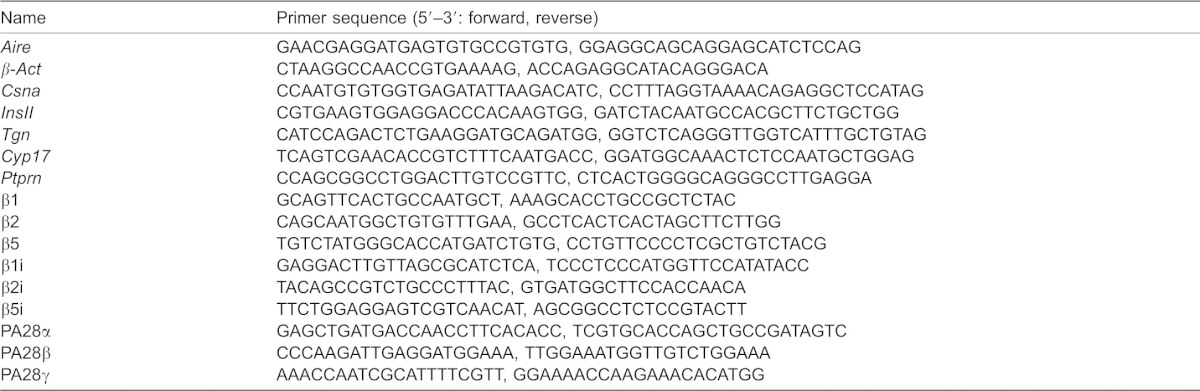
Primer sequence of Aire, TSA and proteasome for qRT-PCR analysis

**Table 2.**
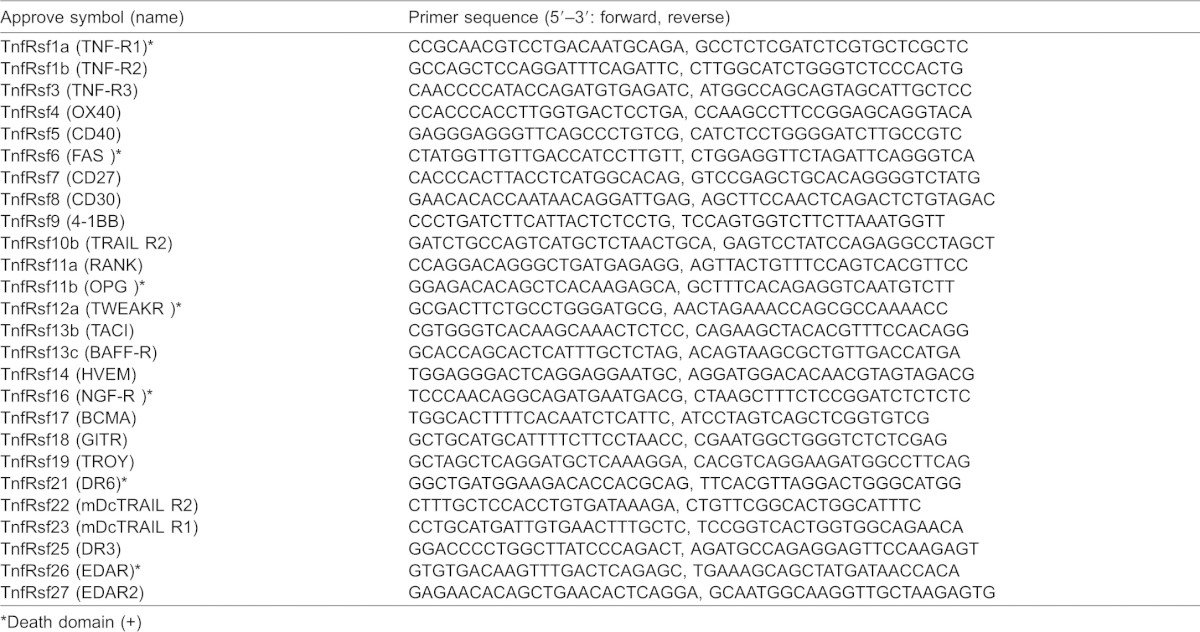
Primer sequence of TnfRsfs for qRT-PCR analysis

### Antibodies and western blotting

Anti-mouse Aire protein antibody (anti-Aire-pAb): The synthetic peptides corresponding to the amino acid sequence 126–140 (PPRPPTKRKALEEPR) and 541–522 (DDSRPLAETPPFSS) of mouse Aire protein were conjugated with KLH, and used for immunizing mice (A&G Pharmaceutical Inc.). The primary antibodies used include: Mouse anti β-Actin antibody (Millipore) the mouse Aire-pAb. IRDye 800CW-conjugated Goat-anti-mouse IgG (H+L) (LI-COR) was used as second antibody. For western blotting, cells were lysed in 1% SDS-sample buffer and clarified by centrifugation. Protein concentration of cell lysate was determined by DC Protein Assay (BIO-RAD). The protein bands separated on SDS-PAGE were transferred onto PVDF membranes. Aire protein was detected with anti-Aire-pAb and visualized by ODDYSEY imaging system (LI-COR).

### Separation of thymocytes sub-classes

Thymus was dissected from BDF1 mouse at age of 3–5 weeks (Oriental Yeast Co., Ltd.), cut into small (1 mm) pieces, mashed by scraping with two sterile slide-glasses, and suspended in DMEM containing 10% FBS and penicillin/streptomycin. They were passed through pre-separation filter (Miltenyi Biotech) at 4°C, pelleted by centrifugation at 1500 rpm and re-suspended in DMEM. Those fresh thymocytes (bulk) were fractionated into four sub-classes regarding expression pattern of surface markers CD4 and CD8: CD4^+^CD8^−^ thymocytes, CD4^−^CD8^−^ thymocytes, CD4^−^CD8^+^ thymocytes and thymocytes without CD4^−^. Separation was performed by MACS Separator (Miltenyi Biotech) using antibody-linked magnet beads such as rat-anti-mouse-CD4MicroBeads and rat-anti-mouse-CD8/MicroBeads (Miltenyi Biotech).

### Co-culture of Aire^+^ cells with thymocytes or PBLs of normal and Aire-KO (Aire^−/−^) mouse

Bulk thymocytes (BDF1; C57BL/6×DBA/2, 6×10^6^ cells) were co-cultured with Aire^+^ cells (BDF1, 3×10^4^ cells). Fractionated thymocytes (1×10^5^ CD4^−^CD8^−^, 1.2×10^5^ CD4^+^CD8^−^, 1.2×10^5^ CD4^−^CD8^+^ and 6.6×10^6^ thymocytes without CD4^−^) were also co-cultured with Aire^+^ cells. They were incubated for 16 hours in 4-well culture slides (BD Falcon) with DMEM containing 10% FBS and penicillin/streptomycin. Fresh PBL was drained from normal mouse (C57BL/6) and Aire-KO mouse (Aire^−/−^, C57BL/6), a generous gift from the late Professor Leena Peltonen (Ramsey et al., 2002). They were purified by 40–70% Percoll density gradients. The purified PBLs (0.5×10^6^ cells) were co-cultured with Aire^+^ cells (3×10^4^ cells) for 16 hours in DMEM containing 10%FBS and penicillin/streptomycin.

### TUNEL method for apoptosis

After 16 hours or 24 hours cultivations, the free thymocytes were removed by gentle decantation and washed out with cold PBS. The co-cultured cells on 4-well culture slides were fixed with 4% paraformaldehyde in PBS (pH 7.4) for 15 min on ice, and treated with a MEBSTAIN Apoptosis Kit Direct (MBL). The specimens treated with TUNEL method were examined under a confocal fluorescence microscope (ECLIPSE E800, Nikon).

### MTT assay for cell viability

Aire^+^ cells were inoculated into 96-well plates and incubated overnight, then bulk thymocytes were added and co-cultured for 24 hours. As control, the same number of Aire^+^ cells and thymocytes were cultured into separate wells. Viable cells were counted by measuring absorbance at 570 nm (A570) after staining with MTT (Roche).

### Flow cytometry analysis

Aire^+^ cells were grown to semi-confluency or confluency in RepCell culture dish (CellSeed). Aire^+^ cells were detached by gentle pipetting at 20°C. The detached cells were washed once with cold PBS and fixed with 4% paraformaldehyde in PBS (pH 7.4) for 15 min on ice. Then, Aire^+^ cells were stained with fluorescent antibodies specific for each of IS components CD40 (Alexa Fluor 647 anti-mouse CD40, BioLegend), MHC-II (Alexa Fluor 488 anti-mouse I-A/I-E, BioLegend), CD80 (Alexa Fluor 488 anti-mouse CD80, BioLegend), CD86 (Alexa Fluor 647 anti-mouse CD86, BioLegend), and control IgG. After washing with cold PBS, surface fluorescence of the stained Aire^+^ cells (5,000 cells each) was measured by a flow cytometry system Guava (Millipore).

## RESULTS

### All three Aire^+^ cell lines (Aire^+^TEC1, Aire^+^TEC2 and Aire^+^DC) consistently express endogenous Aire protein

**Fig. 1.**
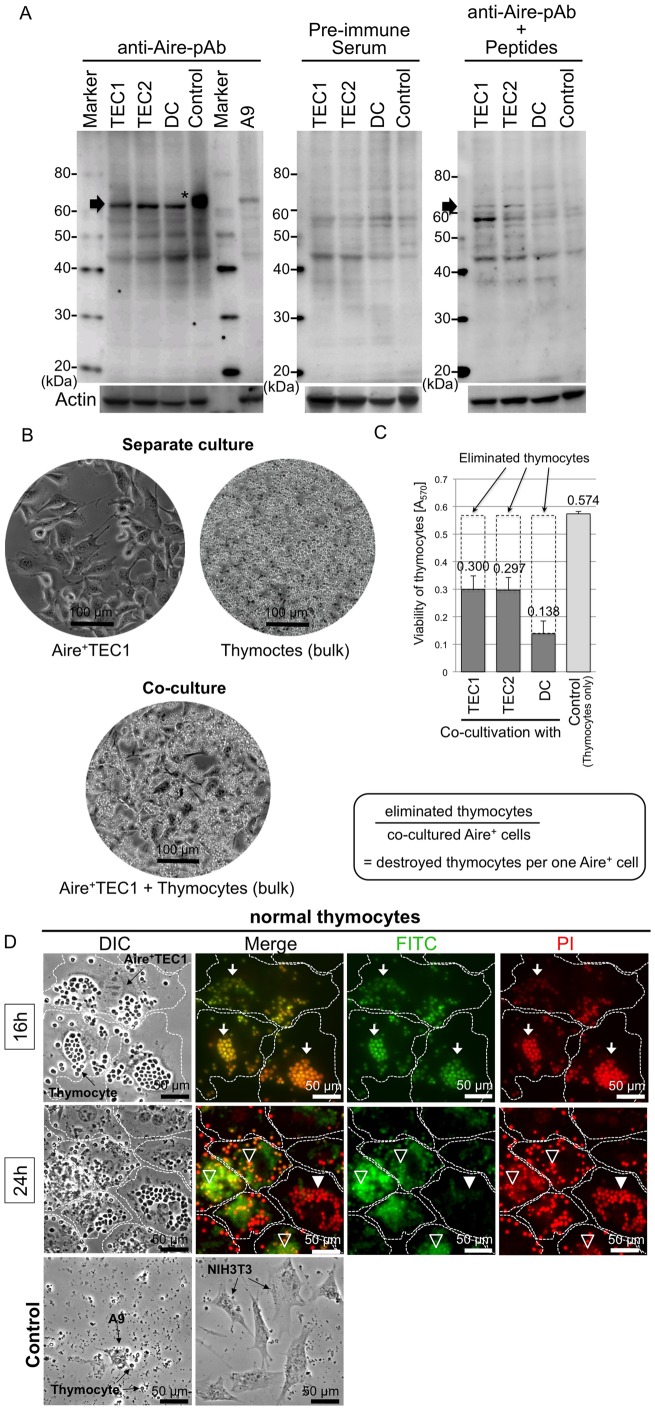
Detection of endogenous Aire protein and observation of *in vitro* some aspect for negative selection of thymocytes *in vivo* by three lines of Aire^+^ cells. (A) Detection of mouse endogenous Aire proteins with anti-Aire antibody. Aire^+^ cell lysates were electrophoresed on 5–20% gradient polyacrylamide gel and processed for Western blotting with mouse polyclonal antibody (anti-Aire-pAb). Arrow indicates the location of Aire-protein (Mr = 62 kDa) along protein markers. Asterisk in Control indicates the FLAG-Aire fusion protein that was overexpressed in the transformed Aire^+^DC. In A9 cell line used as negative control, target size band was not detected. The anti-Aire-pAb pre-absorbed with synthetic mouse Aire peptides and pre-immune serum were used to confirm immune specificity. (B) Microscopic observation of co-cultures and separate cultures. Fresh thymocytes (7.0×10^6^) and Aire^+^ cells (7.5×10^4^ for Aire^+^TEC1 and TEC2, 3.0×10^5^ for Aire^+^DC) were grown for 24 hrs as separate cultures or co-culture. Almost equal numbers of living Aire^+^ cells were counted in the separate culture and co-culture (59 and 61 cells in a visual field). Scale bar, 100 µm. (C) Estimation of viable cell number. Aire^+^ cells and the equivalent numbers of thymocytes were inoculated and cultured for 24 hrs, then living cells were stained with MTT and absorbance at 570 nm (A_570_) was measured. The cells used were: TEC1 and TEC2 (2.76×10^4^ and 2.88×10^4^), DC (9.60×10^4^) and thymocytes (2.58×10^6^). Average of four separate experiments was shown with standard deviation (bar). The reduction of absorbance value directly indicates the number of killed thymocytes. Note: significant reduction of thymocytes (dashed line regions) in contrast to thymocytes only. The number of eliminated thymocytes was estimated using A_570_ and Aire^+^ cell number. It was calculated that within 24 hrs single Aire^+^ cell appeared able to kill ∼30 thymocytes i*n vitro*. (D) Apoptosis and non-apoptosis of fresh thymocytes induced by Aire^+^ cells: Fresh thymocytes (7.0×10^6^) were added into the culture of Aire^+^TEC1 (7.5×10^4^) and incubated for 16 h and 24 h. DNA fragmentation as an indication of apoptosis was detected with FITC (green) by TUNEL method and nuclear DNA was stained with PI (red). The wavy broken lines indicate the outline of Aire^+^TEC1 detected under DIC. Scale bar, 50 µm. [16 h] White arrows indicate the clusters of apoptotic thymocytes adhering on Aire^+^TEC1 (stained with both FITC and PI). [24 h] Open arrowheads indicate the clusters of apoptotic thymocytes (stained with both FITC and PI), under which smashed thymocytes on membrane of Aire^+^TEC1 are also stained with FITC. A white arrowhead indicates a cluster of thymocytes that seem alive (stained with PI but not with FITC) while tightly adhering on a living Aire^+^TEC1. NIH3T3 and A9 cells were used as control, and no adherence with thymocytes was observed.

In our initial characterization study (Yamaguchi et al., 2011), expression of Aire mRNA was clearly detected in all three Aire^+^ cell lines (Aire^+^TEC1, TEC2 and DC) but endogenous Aire protein could not be detected, perhaps due to poor quality of polyclonal antibodies used. In the present study, we succeeded to produce a new polyclonal anti-Aire antibody (pAb) by immunizing mice with a mixture of synthetic peptides, which correspond to two peptide fragments (126–140 aa and 541–552 aa) of mouse Aire protein. The resulting pAb clearly detected endogenous mouse Aire proteins of Mr = 62 kDa on Western blots in all three Aire^+^ cell lines (Aire^+^TEC1, Aire^+^TEC2 and Aire^+^DC). Also, FLAG-tagged Aire protein was detected (see arrowed three bands and an asterisked band in Fig. 1A, leftside panel). Pre-immune serum or antibodies pre-absorbed with synthetic peptides detected no protein band of Mr = 62 kDa (Fig. 1A, middle and right-side panel). Thus, the newly made anti-Aire pAb had proper immune specificity. Moreover, the protein band of Mr = 62 kDa was not detected in mouse A9 skin fibroblasts. Thus, endogenous Aire protein was detected in all three Aire^+^ cell lines.

### A single Aire^+^ cell appeared able to kill ∼30 thymocytes

Previously, we demonstrated that Aire^+^ cells adhere to fresh thymocytes and kill them by inducing apoptosis (Yamaguchi et al., 2011). Here, we examined how efficiently those Aire^+^ cells kill thymocytes using our *in vitro* co-culture system. For this, dissected mouse (strain BDF1) thymus was smashed and dissociated thymocytes were filtered through membrane. They were then added into culture plates of Aire^+^ cells or maintained as separate cultures. After incubation for 24 hrs, culture plates were carefully examined under a phase-contrast microscope (Fig. 1B, without the removal of free thymocytes). In the separate culture as control, Aire^+^ cells and thymocytes (bulk) were maintained without significant loss of cell number. However, in the co-culture system (see Aire^+^TEC1 + thymocytes in Fig. 1B), thymocytes (bulk) were substantially reduced while Aire^+^ cells remained unchanged (note cell density difference between separate culture and co-cultures in Fig. 1B).

Direct estimation of “killed thymocytes” is impossible because they are destroyed by apoptosis within 24 hrs. So, we estimated surviving viable cells by measuring absorbance at 570 nm (A570) after MTT-color reaction. Only live cells can change MTT to insoluble formazan and generate blue color at 570 nm. Culture medium alone never affected MTT-color reaction. For all co-cultures, total absorbance at A_570_ (cell viability) was significantly reduced (20∼25%) whereas absorbance of separate cultures remained unchanged (Fig. 1C), indicating significant cell loss in the co-cultures. Under closer microscopic observation, almost equal numbers of live Aire^+^ cells were counted in a small field for both separate culture and co-culture (59 cells *vs* 61 cells), so that reduction of absorbance at A_570_ (20∼25%) was correlated to killed thymocytes (Fig. 1C). Since absorbance of thymocytes in separate culture was 0.574 (2.58×10^6^ cells), the absorbance difference from Aire^+^TEC1 (A_570_ = 0.300; 2.76×10^4^ cells), TEC2 (A_570_ = 0.297; 2.88×10^4^ cells) and DC (A_570_ = 0.138; 9.60×10^4^ cells) was calculated as 0.274 (1.23×10^6^ thymocytes), 0.277 (1.25×10^6^ thymocytes) and 0.436 (1.96×10^6^ thymocytes), respectively. Then, live cell numbers were calculated as indicated in Fig. 1C legend. Considering both absorbance reduction and live cell number, we calculated that a single Aire^+^ cell killed 20∼45 thymocytes: 44.5 thymocytes for Aire^+^TEC1, 43.2 thymocytes for Aire^+^TEC2 and 20.4 thymocytes for Aire^+^DC. On average, a single Aire^+^ cell appeared able to kill ∼30 thymocytes.

### Some thymocytes escape from Aire^+^ cell-dependent apoptosis (elimination)

So far, we demonstrated *in vitro* adherence of fresh thymocytes to Aire^+^ cells and induction of apoptosis. More careful microscopic examination on co-culture revealed that after 16 hrs incubation some thymocytes are still attached on Aire^+^ cell surface (see Aire^+^TEC1 cells in Fig. 1D, with the removal of free thymocytes). TUNEL assay stained these thymocytes green with FITC (green), suggesting they may be in the process of apoptosis (going to die) (see three white arrows in FITC panel of Fig. 1D, 16 h). Most of them clumped on Aire^+^ cells (see three white arrows in PI panel of Fig. 1D, 16 h, also see Merged yellow image). They still retained nuclei (stained with PI: red) and most of them formed clumps on Aire^+^ cell surface (see three white arrows in PI panel of Fig. 1D, 16 h, also see Merged yellow image). On the contrary, Aire^+^TEC1 cells were not stained with TUNEL assay (surrounded by four dashed lines). Thus, FITC-stained (apoptotic) thymocytes attached on Aire^+^ cell surface are still intact at 16 hrs.

After longer cultivation (24 hrs), many Aire^+^TEC1 cells seemed stained with FITC (green) (note Aire^+^TEC1 cells that are seen under thymocytes marked with three open arrowheads in FITC panel of Fig. 1D, 24 h). We assume that fluorescence may have come from the cell debris of destroyed thymocytes (see also Merged yellow–green image). Thus, apoptosis-induced thymocytes were broken after 24 hrs. However, a large clump of thymocytes still adhering to Aire^+^TEC1 cell (marked with a closed white arrowhead in Fig. 1D, 24 h) was not stained with FITC, indicating that these thymocytes are not susceptible to Aire^+^ cell-induced apoptosis. Large clumps of non FITC-stained thymocytes are often observed in co-culture. As control, mouse skin fibroblast A9 and embryonic fibroblast NIH3T3 showed no adherence to thymocytes (Fig. 1D, Control).

In short, apoptotic process of thymocytes is induced within 16 hrs and auto-reactive thymocytes are destroyed during 16–24 hrs. Moreover, some thymocytes can escape from Aire^+^ cell-induced apoptosis (elimination).

### Aire^+^ cells can kill “auto-reactive PBLs” that are abundant in Aire-KO (Aire^−/−^) mouse

**Fig. 2.**
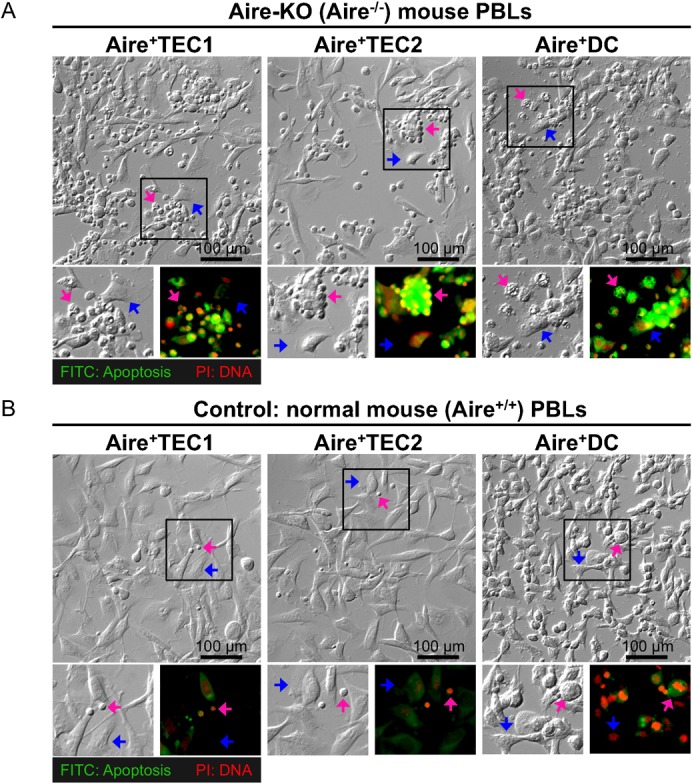
Aire^+^ cells are able to trap “auto-reactive PBLs” both from Aire-KO (Aire^−/−^) mouse and normal (Aire^+/+^) mouse as control, and the trapped PBLs are induced for apoptosis. (A) Apoptosis of Aire-knockout (Aire^−/−^) mouse PBLs: Blood was drawn from femoral blood vessels. The PBLs were separated by Percoll gradients. After washing with culture medium, viability of PBLs was confirmed by trypan blue staining, fresh PBLs (5×10^6^) were added into the culture of Aire^+^ cells (7.5×10^4^ for Aire^+^TEC1 and TEC2, 3.0×10^5^ for Aire^+^DC) and incubated for 24 h. Later, free PBLs were removed from the dishes by gentle decantation and washed three more times with PBS. DNA fragmentation was detected with FITC (green) by TUNEL method and nuclear DNA was stained with PI (red). Scale bar, 100 µm. (B) Apoptosis of normal (Aire^+/+^) mouse PBLs: Blood was drawn from femoral blood vessels: Co-cultures with Aire^+^cells and TUNEL/PI stains were performed as mentioned above. Scale bar, 100 µm.

To examine if Aire^+^ cells interact with peripheral blood lymphocytes (PBLs), we performed co-culture experiments using Aire^+^ cells and fresh PBLs prepared from either Aire-KO (Aire^−/−^) mouse or normal (Aire^+/+^) mouse. These freshly drained PBLs were partially purified by Percoll density gradients and added into Aire^+^ cell cultures. After 24 hrs incubation, floating PBLs were carefully removed by gentle decantation (Fig. 2). When PBLs drained from Aire-KO (Aire^−/−^) mouse were tested, abundant PBLs adhered to Aire^+^ cells (Fig. 2A, with the removal of free PBLs). Notably, Aire^+^DC line trapped PBLs more efficiently than two other Aire^+^ cell lines. TUNEL assay stained those PBLs green, indicating that they are in the process of apoptosis (see PBLs marked with magenta arrows in Fig. 2A). In the case of PBLs drained from normal (Aire^+/+^) mouse, only a few PBLs adhered to Aire^+^ cells (Fig. 2B, with the removal of free PBLs). Thus, those PBLs adhered to Aire^+^ cells are considered to be “auto-reactive PBLs”. Aire-KO (Aire^−/−^) mouse had substantially more “auto-reactive PBLs” than normal (Aire^+/+^) mouse since thymus of Aire-KO (Aire^−/−^) mouse lost ability of negative selection.

### *Aire* gene expression increases as cell density increases

**Fig. 3.**
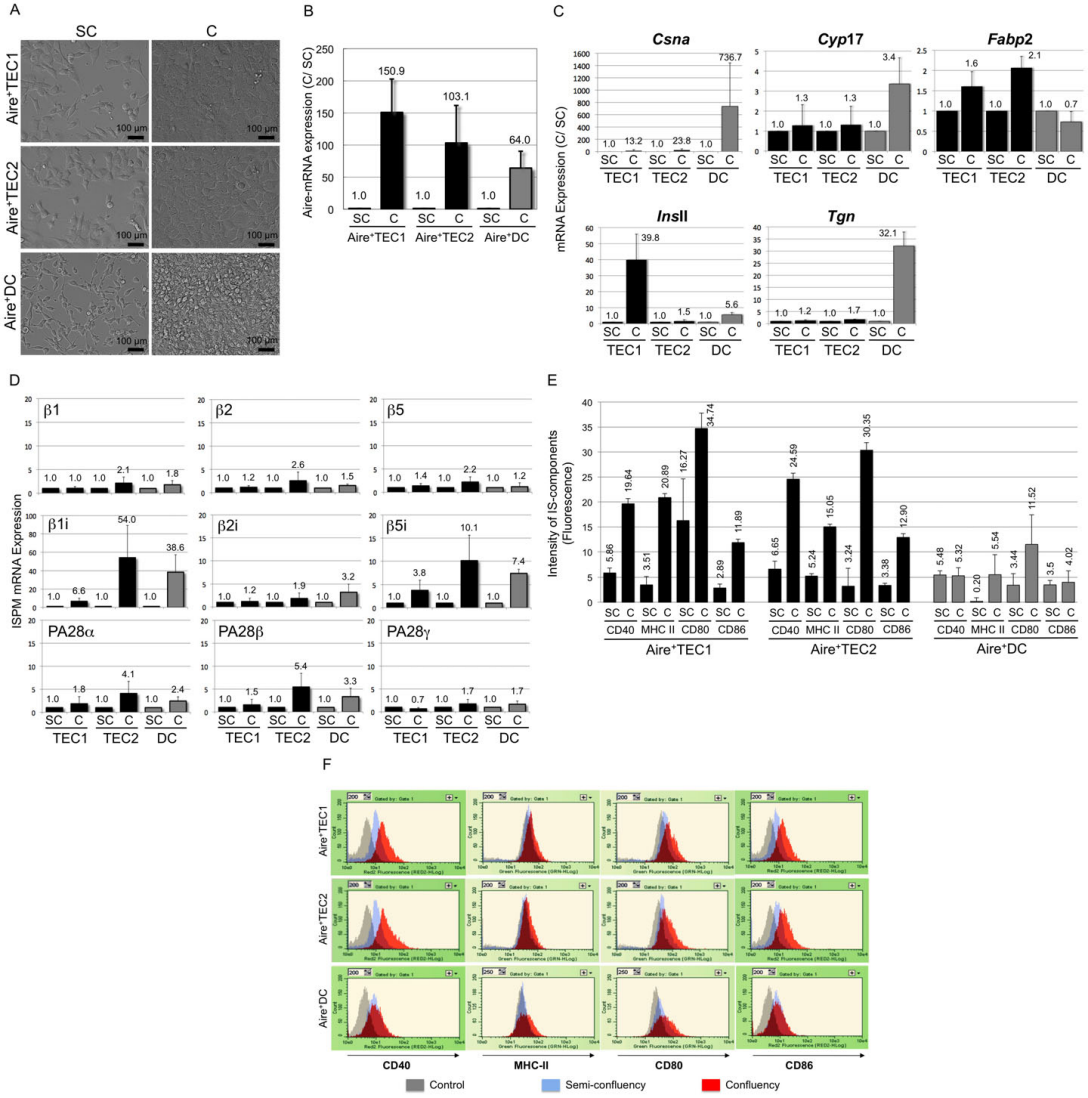
Morphology and comparative analysis of increasing gene expression of Aire, TSAs and components for IS and IPSM in semi-confluent and confluent conditions. (A) Cell morphology. Three Aire^+^ cell lines (TEC1, TE2 and DC) were grown to semi-confluency (SC) (0.75×10^6^ cells/dish for Aire^+^TEC1 and Aire^+^TEC2, 2.0×10^6^ cells/dish for Aire^+^DC) and to confluency (C) (3.5×10^6^ cells/dish for Aire^+^TEC1 and Aire^+^TEC2, 1.4×10^7^ cells/dish for Aire^+^DC). Pictures were taken by a Nikon phase-contrast microscope ECLIPSE Ti-U. Scale bar, 100 µm. (B) Aire-mRNA expression. Aire-mRNA in total RNAs was quantified by qRT-PCR analysis, the amount of Aire-mRNA was normalized to β-Actin and the expression was compared between two cell densities (SC and C). Average of three separate experiments was shown with standard deviation (bar). (C) Expression of TSA-mRNAs. TSA-mRNA in total RNAs was quantified by qRT-PCR analysis with primers specific for 5 different TSAs (Csna, Cyp17, Fabp2, InsII and Tgn) and β-Actin, the amount of TSA-mRNA was normalized to β-Actin and the expression was compared between two cell densities (SC and C). Average of three separate experiments was shown with standard deviation (bar). The expression levels at SC were set as 1.00. Note the scale difference in the vertical axis. (D) Expression of mRNAs for subunits of PSM and IPSM. The subunit (Sub)-specific mRNA in total RNA was quantified by qRT-PCR analysis with primers specific for various subunits of PSM and IPSM (Table 1) and β-Actin. The amount of Sub-mRNA was normalized to β-Actin and the expression was compared between two cell densities (SC and C). Average of three separate experiments was shown with standard deviation (bar). The expression levels at SC were arbitrarily set as 1.00. Note the scale difference in the vertical axis. Upper panel: mRNAs for PSM-subunits (β1, β2, β5), Middle panel: mRNAs for IPSM-subunits (β1i, β2i β5i), Bottom panel: mRNAs for IPSM-subunits (PA28α, PA28β and PA28γ). (E) Expression of IS-components. Aire^+^ cell lines (5,000 cells each) at different cell density (SC and C) were stained with fluorescent antibodies specific to IS-components (CD40, MHC-II, CD80 and CD86) and the surface fluorescence was measured by a flow cytometry system Guava (Millipore). The background fluorescence by the same animal IgG was subtracted. The X-Arithmetic means of four separate experiments were plotted with standard deviation (bar). (F) Flow cytometry analysis of surface markers: Aire^+^TEC1, TEC2 and DC cells at semi-confluent and confluent conditions were analyzed with specific antibodies (CD40, MHC class II, CD80 and CD86) for cell surface marker. Each panel shows the distribution of the markers by fluorescent intensity *vs* cell count. The histograms were compared among control IgG (gray), semi-confluent (blue) and confluent condition (red).

We then examined if cell growth states of Aire^+^ cell affects expression levels of *Aire* gene. To test this, we prepared Aire^+^ cells (Aire^+^TEC1, Aire^+^TEC2 and Aire^+^DC) at two different growth states: rapidly growing state at semi-confluency (SC) and resting state at confluency (C) (Fig. 3). Aire^+^ cells at SC exhibited characteristic shapes of thymic epithelial cells or dendritic cells, whereas all Aire^+^ cells at C formed monolayers of tightly packed cells with cell–cell contact (Fig. 3A).

Unexpectedly, in our preliminary experiments, quantitative Reverse Transcription-PCR (qRT-PCR) analysis showed some fluctuation in expression of housekeeping genes (Gapdh, Hprt1, G6pd2, TATA box-binding protein, γ-Actin and polyubiquitin-C) depending on cell growth states, but expression of β-Actin was consistent (data not shown). Hence, β-Actin was used as a reference gene to compare with *Aire* gene expression, and each Aire mRNA content was normalized to β-Actin mRNA. Then, the normalized Aire-mRNA expression was compared between two different growth states (SC *vs* C). It was found that Aire mRNA at C is always higher than SC (Fig. 3B). The increase was significant (60×∼150×) when compared per cell basis. Based on these findings, we postulated that increase of Aire-mRNA expression occurs in accordance with cell density (cell growth states) and it may mimic *in vivo* developmental process of mTEC (Gray et al., 2007).

### Expression of several tissue-specific antigens (TSAs) increases at higher cell density

Previously we demonstrated that all three Aire^+^ cell lines exhibit ectopic (promiscuous) expression of TSAs (Yamaguchi et al., 2011). It was also reported that Aire-KO (Aire^−/−^) mice exhibit reduced expression of TSAs (Anderson et al., 2002).

These findings suggest critical roles of Aire genes in TSA expression. Here, we further examined possible effects of cell growth stage on ectopic expression of five representative TSAs, including Casein alpha (*Csna*) specific for thyroid, Cytochrome P450 17 (*Cyp*17) specific for gonad, Fatty acid binding protein 2 (*Fabp*2) specific for intestine, Insulin II (*Ins*II) specific for pancreas and Thyroglobulin (*Tgn*) specific for thyroid.

qRT-PCR analysis revealed that these organ-specific TSA genes (*Csna, Cyp*17, *Fabp*2, *Ins*II and *Tgn*) are expressed in all three Aire^+^ cell lines, although expression levels somewhat varied (Fig. 3C). Surprisingly, TSA-mRNA increased (2×∼730×) with increasing cell density (SC *vs* C) (Fig. 3C). Remarkable increase of TSA-gene expression has been seen during developmental stages of Aire-expressing mTECs in mouse thymus (Gray et al., 2007; Gillard and Farr, 2005; Gäbler et al., 2007), hence we postulated that our *in vitro* Aire^+^ cell system may mimic *in vivo* differentiation and maturation process of mTECs and mDCs.

### Expression of immunoproteasome (IPSM) subunits increases at higher cell density

Within antigen-presenting cells (APCs), TSAs are split into small self-antigen peptides through intracellular organelle “immunoproteasome” (IPSM). IPSM is usually re-constructed from constitutive proteasome (PSM) by replacing some subunits with immune types. IPSM plays a crucial role in negative selection of T cells in thymus.

Previously, we observed that all three Aire^+^ cell lines possess IPSMs (Yamaguchi et al., 2011). Here, we measured mRNAs for subunits of IPSM by qRT-PCR analysis and examined if these mRNA contents change at higher cell density (SC *vs* C) (Fig. 3D). Expression of structural subunits (β1, β2 and β5) of constitutive PSM was detected at significant levels but their contents were not much changed with cell growth states (C/SC: 1.0∼2.6) (Fig. 3D, top panel). However, expression of four subunits of IPSM (β1i and β5i for catalytic subunits of 20S, PA28α and PA28β for regulatory subunits) increased significantly toward C (C/SC: 4.1∼54.0) (Fig. 3D, middle and bottom panels). Thus, more PSMs seem converted to IPSMs toward C. These results again confirmed that Aire^+^ cell lines exhibit characteristic natures of self-APCs, and further suggested that enhanced production of IPSM may be occurring in association with increasing expression of TSA genes.

### Expression of four major components of immunological synapse (IS) increases at higher cell density

Promiscuously expressed TSAs are processed to self-antigen peptides within self antigen-presenting cells (self-APCs). Then, these self-antigens are presented to immature thymocytes through MHC molecules with the aid of a bridge “immunological synapse” (IS). IS is composed of CD80, CD86, ICAM1 and so on. And, increased expression of these molecules is considered as indication of the developmental stage of Aire-expressing mTEC. We examined expression of four major components of IS. For this assay, we stained Aire^+^ cells with fluorescently labeled antibodies against CD40, MHC-II, CD80 and CD86. Then, cell surface fluorescence was measured using a flow cytometry system Guava (Millipore). Fluorescence generated by normal control IgGs (Rat IgG for CD40, CD86 and MHC-II and Hamster IgG for CD80) was subtracted. The fluorescence intensity values of Fig. 3E were shown as the subtraction of the measured X-Arithmetic Mean. As shown in Fig. 3E, all three Aire^+^ cell lines expressed IS components. Notably, their expression levels always increased from SC to C. Moreover, Fig. 3F shows surface distribution of IS components, in which each peak shifts to higher intensity (more expression) at C as compared to SC.

### *In vitro* prediction of killer and rescuer thymocytes that may determine the fate of Aire-expressing medullary thymic epithelial cells (mTECs)

It has been reported that single-positive (SP) CD4^+^ thymocytes play critical roles in the development of mTECs in thymus (Irla et al., 2008; Irla et al., 2010). Based on our findings (elevated expression of TSAs and other relevant cellular parameter), we assumed that Aire^+^ cell lines might possess some features of differentiating mTECs. If so, they might still be able to interact with some specialized thymocytes *in vitro*. Such a “thymic crosstalk” is a reciprocal intercellular communication that controls development of both thymocytes and thymic stromal compartment.

**Fig. 4.**
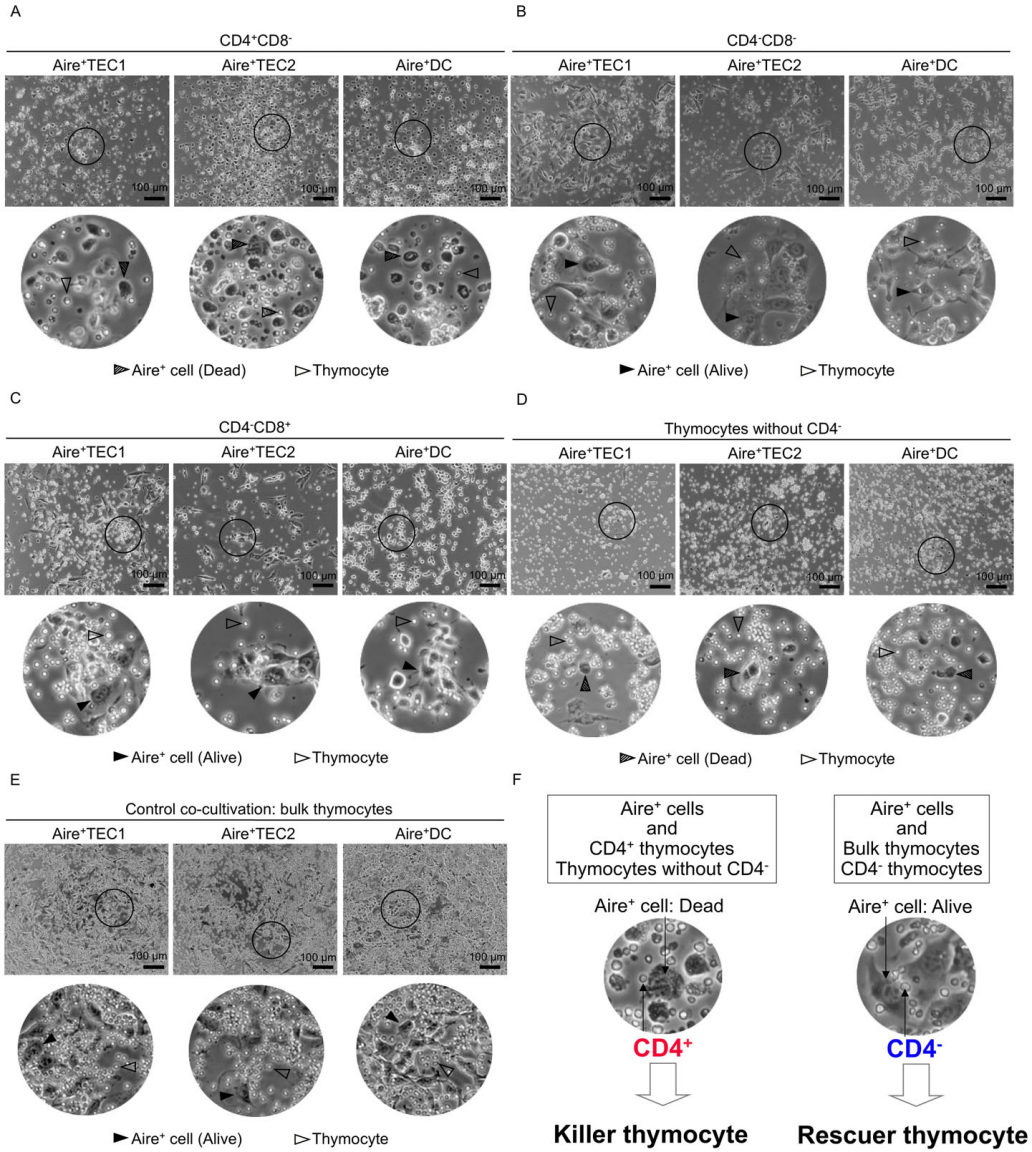
Microscopic observation on the killing of Aire^+^ cels by fractionated sub-populations of thymocytes in our co-culture system. Three Aire^+^ cell lines (Aire^+^TEC1, Aire^+^TE2 and Aire^+^DC) were co-cultured for 20 hrs together with fractionated sub-populations of thymocytes and examined under a phase-contrast microscope ECLIPSE Ti-U (Nikon). (A) Aire^+^ cell lines co-cultured with CD4^+^CD8^−^ thymocytes: Light-reflecting small round cells are thymocytes. In the magnified photos, Aire^+^ cells are seen as disrupted dead cells (shaded arrowhead) among live thymocytes (open arrowhead). These dead Aire^+^ cells were easily washed off with PBS. Scale bar, 100 µm. (B) Aire^+^ cell lines co-cultured with CD4^−^CD8^−^ thymocytes: Aire^+^ cells are alive (black arrowhead) among live thymocytes (open arrowhead). (C) Aire^+^ cell lines co-cultured with CD4^−^CD8^+^ thymocytes: Aire^+^ cells are alive (black arrowhead) among live thymocytes (open arrowhead). (D) Aire^+^ cell lines co-cultured with the thymocytes without CD4^−^. Aire^+^ cells are dead (shaded arrowhead) among live thymocytes (open arrowhead). Dead Aire^+^ cells were easily washed off with PBS. (E) Aire^+^ cell lines co-cultured with the bulk thymocytes containing CD4^+^ and CD4^−^ thymocytes: Aire^+^ cells are apparently alive (black arrowhead) with live thymocytes (open arrowhead). (F) A summary showing that the fate (**dead** or alive) of Aire^+^ cells may be determined by two sub-types of thymocytes. Aire-expressing cells *in vivo* are derived from mTECs and mDCs, which are at intermediate stages of differentiation and maturation. Our *in vitro* co-culture system implies that two novel sub-types of thymocytes (CD4^+^ killer and CD4^−^ rescuer) may exist to determine the fate (dead or alive) of Aire-expressing mTECs (see text in detail).

To examine this possibility *in vitro*, we first prepared four sub-populations of thymocytes: CD4^+^ SP thymocytes (CD4^+^CD8^−^), CD8^+^ SP thymocytes (CD4^−^CD8^+^), CD4^−^CD8^−^ DN thymocytes, and the thymocytes without CD4^−^. Fractionation was done by separating fresh thymocytes using antibody-linked magnetic beads. Then, we performed co-culture experiments (Fig. 4).

To our surprise, when three lines of Aire^+^ cells were co-cultured with CD4^+^ SP (CD4^+^CD8^−^) thymocytes, almost all Aire^+^ cells were killed (Fig. 4A, upper panel). At closer look, shape of floating Aire^+^ cells changed to spherical (Fig. 4A, lower panel, shaded arrowheads) and those were easily removed by gentle PBS-washing. On the contrary, when Aire^+^ cells were co-cultured with CD4^−^ thymocytes (CD4^−^CD8^−^ DN and CD4^−^CD8^+^), all Aire^+^ cells remained alive (Fig. 4B,C, closed arrowheads). Surprisingly, when Aire^+^ cells were co-cultured with thymocytes without CD4^−^ thymocytes, Aire^+^ cells were killed again (Fig. 4D, shaded arrowheads). Thus, *in vitro* killing of Aire^+^ cells by CD4^+^ thymocytes (CD4^+^CD8^−^ and thymocytes without CD4^−^) was demonstrated. Killing of Aire^+^ cells were not obviously seen when we used bulk thymocytes that contain all CD4^+^/CD4^−^ sub-types (Fig. 4E, closed arrowheads). This may be accounted for by the notion that killing or rescuing Aire^+^ cells are minute events and can be seen only when co-cultured with condensed sub-populations of thymocytes.

Aire-expressing cells *in vivo* are derived from mTECs and mDCs that are at intermediate stages of differentiation and maturation. Hence, our *in vitro* observation implied possible existence of two sub-types of thymocytes that may determine the fate (**dead** or alive) of Aire-expressing mTECs. One type is “CD4^+^ killer” thymocytes, which we assume equivalent to CD40L/RANKL CD4^+^ thymocytes. The other type is a novel “CD4^−^ rescuer” thymocytes that may prevent Aire-expressing mTECs/mDCs from the elimination (Fig. 4F).

### Expression of tumor necrosis factor-receptor super families (TNFRSFs)

It has been reported that tumor necrosis factor-receptor super families (TNFRSFs) with “death domain” are involved in the induction of cell death (Hehlgans and Pfeffer, 2005). Previously, we observed the expression of some TNF family members in all three Aire^+^ cell lines (Yamaguchi et al., 2011). Here, we examined if Aire^+^ cell lines express their receptors (TNFRSFs).

**Fig. 5.**
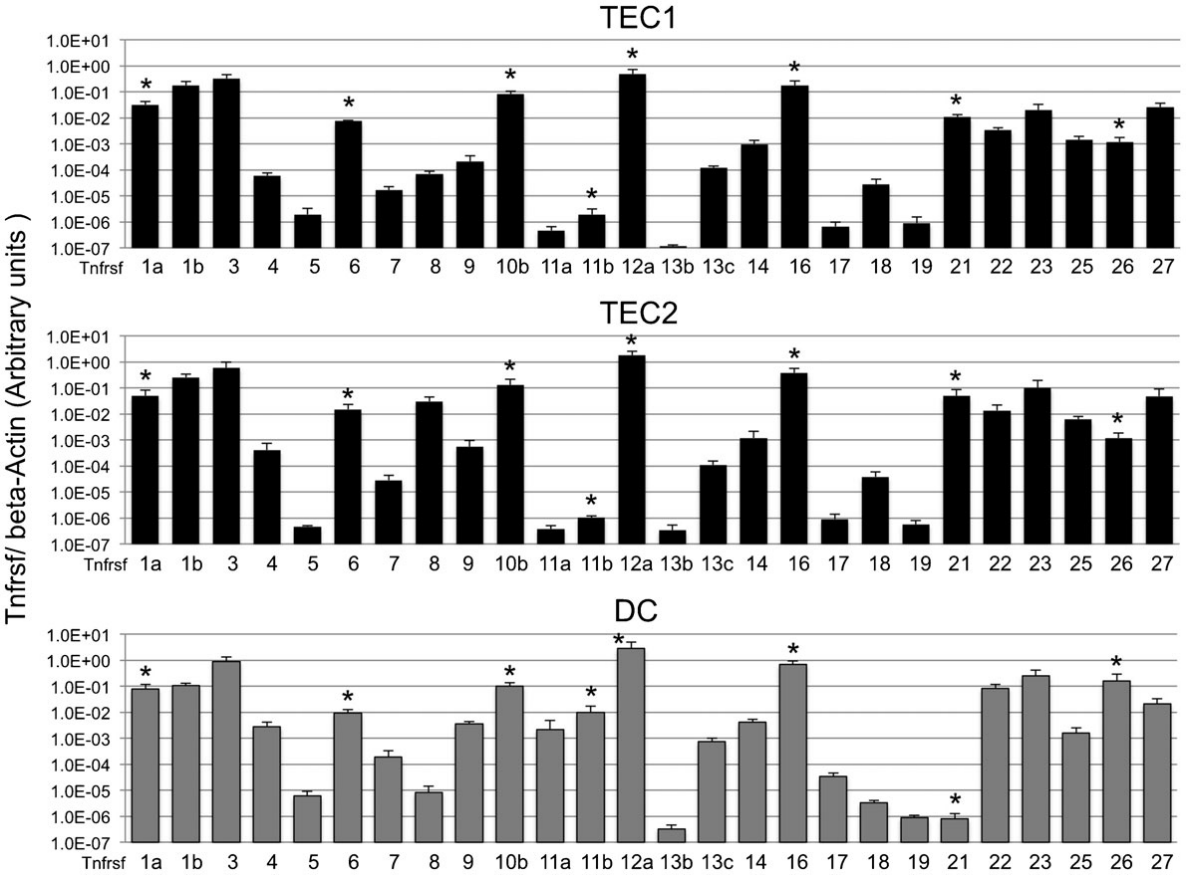
Expression of several genes specific for tumor necrosis factor-receptor super families (TNFRSFs) with and without death domain in three Aire^+^ cell lines. The Tnfrsf-specific mRNA in total RNA was quantified by qRT-PCR analysis with primers specific for 26 different Tnfrsf members (Tnfrsf-1a, -1b, -3, -4, -5, -6, -7, -8, -9, 10b, -11a, -11b, -12a, -13b, -13c, -14, -16, -17, -18, -19, -21, -22, -23, -25, -26 and -27) and β-Actin. Values are arbitrary units (a.u.) normalized to β-Actin. Average of three separate experiments was shown with standard deviation (bar) above the mean of at least three independent experiments. Asterisk indicates eight members known to possess death domain (Tnfrsf-1a, -6, -10b, -11b, -12a, -16, -21, and -26).

qRT-PCR analysis revealed that a number of TNFRSF members are expressed in all three Aire^+^ cell lines (Fig. 5). Most of 26 members tested were expressed at similar levels. However, expression of five members (Tnfrsf-8, -11a, -11b, -17, -21) was different between two cell types (Aire^+^TECs and Aire^+^DC). On the other hand, no difference was seen for other five members (Tnfrsf-1a, -6, -10b, -12a, and -16).

Among 26 members examined, eight members (Tnfrsf-1a, -6, -10b, -11b, -12a, -16, -21, and -26) are known to possess “death domain” (Hehlgans and Pfeffer, 2005; Tartaglia et al., 1993; Itoh and Nagata, 1993; Pan et al., 1997; Yamaguchi et al., 1998; Chicheportiche et al., 1997; Chao, 1994; Pan et al., 1998; Simonet et al., 1997). Considering the apoptotic ability of death domain-containing TNFRSFs, we assumed that five TNFRSF members (Thfrsf-1a, -6, -10b, -12a and -16) may be involved in killing Aire^+^ cells. Thus, these *in vitro* results seem to support the TNF/TNFRSF-mediated self-killing of Aire^+^ mTECs.

## DISCUSSION

AIRE gene has been cloned from human chromosome 21 as a pathogenic gene responsible for autoimmune disease APECED/APS-1 that exhibits autosomal recessive inheritance (Nagamine et al., 1997). In human and mouse, it was shown that AIRE gene expression is restricted to only the immune system such as thymus and lymph node, and that Aire protein acts as a unique transcription factor (Nagamine et al., 1997; Pitkänen et al., 2000; Pitkänen et al., 2001). Furthermore, several studies using Aire-deficient mutant mice provided an important evidence that transcription factor Aire plays a critical role in regulating the ectopic (promiscuous) expression of tissue-specific antigens (TSAs) in the mTECs of thymus (Anderson et al., 2002; Ramsey et al., 2002; Liston et al., 2003).

Recently, we established three lines of Aire-expressing cells (Aire^+^TEC1/2 and Aire^+^DC) from abnormally enlarged thymus of the transgenic mice which were produced by injecting a transgene consisting of SV40 T-antigen (SV40-Tag) gene driven by mouse Aire gene promoter into fertilized eggs (Yamaguchi et al., 2011). SV40-Tag protein binds to tumor suppressors p53 and retinoblastoma family (pRb, p107 and p130) that are involved in regulation of cell cycle. Interaction of these proteins with SV40-Tag facilitates cell proliferation and survival, hence by transfecting with SV40-Tag gene a number of transformed cell lines have been established (Gannon and Lane, 1987; Bártek et al., 1992; Ishida et al., 1995; Yanai et al., 1991).

In our transgenic mice, SV40-Tag gene was driven by Aire-gene promoter, hence its expression was allowed only in the cells like mTECs expressing Aire. In all three established Aire^+^ cell lines, endogenous Aire protein was undoubtedly detected (Fig. 1A). To date, a large quantity of Aire-expressing mTECs have never been isolated from mouse thymus, hence newly isolated three Aire^+^ cell lines are only available ones and will be extremely useful for *in vitro* studying biological aspects of AIRE protein functions at molecular levels.

All three Aire^+^ cells adhered to fresh thymocytes *in vitro* and induced apoptosis, potentially reproducing *in vivo* T-cell negative selection occurring in the thymus medulla. Moreover, Aire^+^ cells exhibited characteristic features of “self-APCs”, such as abilities to express TSAs (tissue specific antigens), IPSM (immunoproteasome), IS (immunological synapse) and TNFs/TNFRSFs (tumor necrosis factors/TNF receptors). Thus, Aire^+^ cell lines were considered as “self-APCs” acting *in vitro*.

Using our *in vitro* co-culture system, it was calculated that a single Aire^+^ cell appeared able to kill 20–45 (∼30) thymocytes within 24 hrs (Fig. 1B,C). Interestingly, two Aire^+^TEC lines killed thymocytes more efficiently than Aire^+^DC line, probably reflecting expression patterns of TSAs (self-antigens) in those Aire^+^ cells.

A major event occurring in thymus medulla is elimination of auto-reactive thymocytes (T cells). This is critical to secure self-tolerance in immune system. In thymus, auto-reactive thymocytes are apoptosed *via* “negative selection”. As documented above, Aire^+^ cells induce apoptosis on adhered thymocytes (Yamaguchi et al., 2011). In the co-culture system, TUNEL assay indicated that apoptosis occurs after 16 hrs and DNA fragmentation takes place within 24 hrs (Fig. 1D). Non-thymic cells (mouse fibroblasts NIH3T3 and A9) did not adhere to any thymocytes. Interestingly, some thymocytes were not stained with TUNEL assay (not killed by Aire^+^ cells), suggesting that these minute thymocytes may be “non auto-reactive thymocytes” that will be released into peripheral blood.

In patients with autoimmune disease, it was shown that auto-reactive T cells escape from negative selection in thymus and are drained into peripheral blood (Perniola et al., 2000). Those escaped T cells injure several tissues/organs and disrupt immune tolerance. Such aberrant T cells are found also in peripheral blood lymphocytes (PBLs) of health body, though a minute fraction (1–5%). This peripheral tolerance is mediated through regulatory T cells.

In our *in vitro* co-culture system, Aire^+^ cells trapped some of fresh PBLs from normal (Aire^+/+^) mouse, and many more from Aire-deficient mutant (Aire^−/−^) mouse (Fig. 2A,B). These Aire^+/+^ and Aire^−/−^ PBLs trapped by Aire^+^ cells were subject to apoptosis. Thus, all Aire^+^ cell lines are capable of trapping “auto-reactive PBLs” that are rarely present in PBLs of normal (Aire^+/+^) mouse and abundant in PBLs of Aire-KO (Aire^−/−^) mouse. These findings suggest a possible use of Aire^+^ cells to absorb/trap auto-reactive PBLs in APECED mouse model. Similarly, once human type Aire^+^ cells were developed, they could be used as absorber/trapper to treat patients with autoimmune disease such as APECED/APS1.

Terminal differentiation model of mTECs (Gray et al., 2007; Gillard and Farr, 2005; Irla et al., 2010) postulates that mTECs gradually increase expression of several differentiation-related genes during maturation process in coordination with “thymic crosstalk”. These genes/products include a unique transcription factor “Aire”, a variety of TSAs and essential components of immunological synapse (IS) (MHC-II, CD80, CD86, CD40).

In our *in vitro* Aire^+^ cell culture system, it was shown that expression of Aire gene and organ-specific TSA genes (*Csna, Cyp*17, *Fabp*2, *Ins*II and *Tgn*) increases as cell density increases (Fig. 3A–C). Furthermore, formation of IPMS- and IS-components increased toward confluency (Fig. 3D–F).

Proteasome (PMS) is a protein complex composed of multiple subunits such as a large subunit (20S-particle) with proteolytic activity and three other subunits (19S-particle, 11S (PA28)-particle and PA200) with regulatory roles (Whitby et al., 2000). In self-APC cells, several of these PMS-subunits are replaced with immune-specific counterparts and reconstructed immune type PMSs (IPSMs) act as a processing machinery by which a variety of TSAs (self-proteins) are digested to small peptides (auto-antigens) (Kloetzel and Ossendorp, 2004; Groettrup et al., 2010). Then, these auto-antigens are bound to MHC molecules expressed on the surface of self-APCs. Then, multiple auto-antigens are presented to a specific type of thymocytes.

In our *in vitro* system, all three Aire^+^ cell lines remarkably increased expression of IPSM-specific subunits, such as structural subunits (β1i, β2i and β5i) by ∼50× and regulatory subunits (PA28α, PA28β and PA28γ) by ∼6× toward reaching confluent state (Fig. 3D). Thus, increased IPSMs must be contributing to processing of TSAs to auto-antigen peptides within Aire^+^ cells.

Immunological synapse (IS) is required for mediating the binding of self-APCs to T cells (thymocytes). In our system, Aire^+^ cells expressed four IS components (MHC-II, CD80, CD86, CD40) and their expression levels increased at higher cell density. Thus, increased IS expression on Aire^+^ cells must be enforcing the binding of self-APCs to T cells (thymocytes) (Fig. 3E,F).

These results suggest that remolding of PSM to IPSM and formation of IS must be taking place in the proliferating Aire^+^ cell populations, although molecular studies are required.

Previously, we observed that several kinds of TNFSFs are expressed in all three Aire^+^ cell lines (Yamaguchi et al., 2011). In the present study, we further observed expression of their receptors (TNFRSFs). Interestingly, expression of five TNFRSFs (Tnfrsf-8, -11a, -11b, -17 and -21) was low and shows different patterns between two types of Aire^+^ cell lines (Aire^+^TEC and Aire^+^DC) (Fig. 5). However, six other TNFRSFs (Tnfrsf-1a, -6, -10b, -12a, -16 and -26), that are known to possess death domain (Hehlgans and Pfeffer, 2005; Wiens and Glenney, 2011), showed higher expression levels. Therefore, we presumed that the later six TNFRSF members may play more crucial roles as apoptosis-inducing factors to kill Aire-expressing mTECs. Thus, Aire^+^ cell lines that express TNFSFs/TNFRSFs will serve as useful tools for TNF-associated thymic apoptosis mechanism.

Thymocytes (or T cells) are composed of a variety of sub-types with respect to cell surface markers (CD4^+/−^, CD8^+/−^ and so on). SP-thymocytes are shown to be involved in facilitating maturation process of mTECs such as differentiation, proliferation and survival (Irla et al., 2008; Irla et al., 2010; Akiyama et al., 2008; Hikosaka et al., 2008; Villaseñor et al., 2008; Rossi et al., 2007), but little is known about their regulatory role in governing mature-mTEC development. DP-thymocytes are positively selected by cTECs, and through thymic transport mechanism they are sent to cortico-medullary junction, where DP-thymocytes encounter negative selection conducted by self-APCs.

Our *in vitro* co-culture system demonstrated that CD4^+^ thymocytes kill Aire^+^ cells (Fig. 4A,D), whereas CD4^−^ thymocytes including both CD4^−^CD8^−^ and CD4^−^CD8^+^ are unable to kill Aire^+^ cells (Fig. 4B,C). The sub-population of CD4^+^ thymocytes still contains CD40L^+^/RANKL^+^ thymocytes that are known to apparently determine the fate of Aire-expressing mTECs, and the sub-population of CD4^−^ thymocytes are still heterogeneous as to expression of CD antigens. However, our results implied that some of CD4^+^ and CD4^−^ thymocytes might have distinct ability to determine the fate (**dead** or alive) of Aire^+^ cells, in a similar way as *in vivo* mTECs (Irla et al., 2008; Hikosaka et al., 2008). These include two types of thymocytes; “killer” CD4^+^ thymocytes and “rescuer” CD4^−^ thymocytes (Fig. 4F).

“Killer” CD4^+^ thymocytes killed certain Aire^+^ cells, whereas “rescuer” CD4^−^ thymocytes helped certain Aire^+^ cells from the death. Killing of Aire^+^ cells were not obviously seen when we used bulk thymocytes that contain all CD4^+^/CD4^−^ sub-types (Fig. 4E). This may be accounted for by the notion that killing or rescuing Aire^+^ cells are minute events and can be seen only when condensed sub-populations of thymocytes were used for co-culture.

In short, all three Aire^+^ cell lines increased expression of a number of relevant genes at higher cell density, especially at “confluent” state (Fig. 3B,C,E). These implied that Aire^+^ cell lines progress differentiation stages (from intermediate state to mature state) like mTECs in thymus (supplementary material Fig. S1). Thus, our *in vitro* co-culture system appears to mimic a part of “*in vivo* thymic crosstalk” and would be useful for further studying at molecular basis.

## Supplementary Material

Supplementary Material
