# Supplementary material for: In vitro co-culture systems for studying molecular basis of cellular interaction between Aire-expressing medullary thymic epithelial cells and fresh thymocytes
Source: Biol Open. 2014 Oct 17;3(11):1071–82. doi: 10.1242/bio.201410173 (PMC4232765; doi:10.1242/bio.201410173)
Supplement: Supplementary Material [file supp_3_11_1071__index.html]

In vitro co-culture systems for studying molecular basis of cellular interaction between Aire-expressing medullary thymic epithelial cells and fresh thymocytes — Supplementary Material 

# *In vitro* co-culture systems for studying molecular basis of cellular interaction between Aire-expressing medullary thymic epithelial cells and fresh thymocytes

## bio.201410173 Supplementary Material

**Files in this Data Supplement:**

- Supplementary Material - Yoshitaka Yamaguchi et al. doi: 10.1242/bio.201410173
