# Supplementary material for: In vitro co-culture systems for studying molecular basis of cellular interaction between Aire-expressing medullary thymic epithelial cells and fresh thymocytes
Source: Biol Open. 2014 Oct 17;3(11):1071–82. doi: 10.1242/bio.201410173 (PMC4232765; doi:10.1242/bio.201410173)
Supplement: Supplementary Material [file supp_bio.201410173_bio.201410173-s1.pdf]

Biology Open (2014) 000, 1–12 doi:10.1242/bio.201410173

Supplementary Material

Yoshitaka Yamaguchi et al. doi: 10.1242/bio.201410173

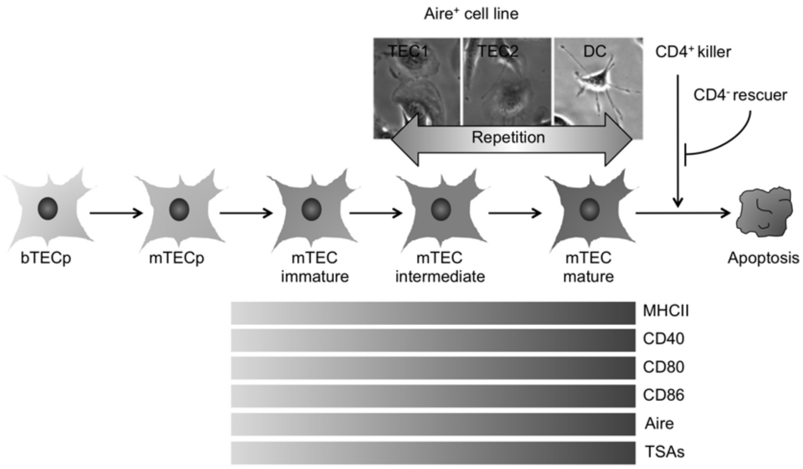

Fig. S1. ***In vitro*** **model of terminal differentiation for Aire$^+$ cells.** Three Aire$^+$ cell lines are shown to mimic the terminal differentiation model of mTEC cells. Expression of a number of differentiation markers gradually increases toward higher cell density while Aire$^+$ cells grow in the culture.
